# Development and characterization of a multifunctional alginate-chitosan hydrogel incorporating nano-hydroxyapatite and *Thymus vulgaris* for wound dressing applications

**DOI:** 10.1016/j.bbiosy.2026.100145

**Published:** 2026-09-03

**Authors:** Kimiya Karimi, Sahebali Manafi, Fatemeh Mirjalili

**Affiliations:** a Department of Materials Engineering, Sha.C., Islamic Azad University, Shahrood, Iran; b Nanoparticle Research Center in Medicine, Sha.C., Islamic Azad University, Shahrood, Iran; c Department of Materials Engineering, May.C., Islamic Azad University, Maybod, Iran

**Keywords:** Alginate-chitosan hydrogel, Hydroxyapatite nanoparticles, Thyme extract, Antimicrobial activity, Wound dressing

## Abstract

Despite significant advances in wound dressing materials, a critical challenge remains in developing a single multifunctional platform that simultaneously provides mechanical integrity, controlled bioactive delivery, and broad-spectrum antimicrobial protection for complex and infected wounds. To address this gap, the present study synthesized and evaluated an innovative alginate-chitosan-based composite hydrogel reinforced with hydroxyapatite nanoparticles (HA) and thyme extract (*Thymus vulgaris*) for advanced wound dressing applications. The primary goal was to create a multifunctional system with optimized mechanical, swelling, biological, and antimicrobial properties. FESEM analyses revealed a uniform distribution and porous morphology in the final sample (Alg/HA/Chi/Ext), while XRD results confirmed a gradual decrease in crystallite size and crystallinity degree from 89.5% for pure HA to 21.7% for the final composite, indicating enhanced amorphous character and intermolecular interactions. Tensile testing demonstrated a significant improvement in the UTS (50 MPa) and, notably, toughness (1087.5 kJ/m³) of the final sample compared to pure alginate hydrogel. This composite exhibited high swelling capacity (766%) similar to pure alginate, but with much better structural stability. The MTT assay confirmed superior biocompatibility and increased cell viability (88% after 72 hours). Release studies showed a biphasic profile for the final sample, which followed the Korsmeyer-Peppas kinetic model. Significantly, this hydrogel demonstrated potent antimicrobial activity against *Bacillus cereus* (98.99% inhibition) and the fungus *Aspergillus flavus* (99.12% inhibition), with very low MIC values (below 5 µg/mL). The developed Alg/HA/Chi/TE hydrogel exhibited significantly enhanced antimicrobial activity (p < 0.05) and superior extract-loading capacity compared with the other formulations, while maintaining controlled release behavior and acceptable biodegradation characteristics. These findings demonstrate the potential of the proposed multifunctional composite as a promising platform for sustained delivery of natural therapeutics in wound-healing applications. Nevertheless, further in vivo studies and sterilization validation are required before clinical translation.

## Highlights

• Novel alginate-chitosan-HA-thyme hydrogel with 50 MPa tensile strength.
• 99% inhibition against B. cereus & A. flavus with MIC <5 µg/mL.
• 88% cell viability and 766% swelling with enhanced structural stability.
• Controlled biphasic release (Korsmeyer-Peppas) & potent antifungal activity.

**Abbreviation**
Alg: Alginate
Chi: Chitosan
HA: Hydroxyapatite
TE: Thyme Extract
TS: Tensile Strength
SR: Swelling Ratio
MIC: Minimum Inhibitory Concentration
MBC: Minimum Bactericidal Concentration
FESEM: Field Emission Scanning Electron Microscopy
FTIR: Fourier Transform Infrared Spectroscopy
XRD: X-Ray Diffraction
UTS: Ultimate Tensile Strength
ROS: Reactive Oxygen Species
GRAS: Generally Recognized As Safe
FWHM: Full Width at Half Maximum
WVTR: Water Vapor Transmission Rate

## 1 Introduction

Hydrogel (HG) dressings represent one of the most important tools in wound management, as they address the essential requirements of an effective wound covering [1,2]. These include maintaining a moist environment while simultaneously absorbing excess exudates, providing a protective barrier that does not adhere to the fragile subcutaneous tissue, reducing pain, and enhancing the overall healing potential [2]. Hydrogel scaffolds function as synthetic extracellular matrices, delivering guiding signals to cells and supporting the formation of new tissue. Owing to their highly hydrated three-dimensional polymeric networks, they closely resemble the structure of the natural extracellular matrix [3]. HGs are a unique class of scaffold with a solid yet gel-like nature, formed by crosslinked hydrophilic polymers that swell substantially in aqueous environments. The space and mechanical stability created by these crosslinks can facilitate the filling of irregular defects caused by injury, thereby promoting the regeneration of functional tissue [4]. A wide variety of natural and synthetic polymers including gelatin, collagen, hyaluronic acid, polyvinyl alcohol (PVA), polyethylene glycol (PEG), and polyacrylamide (PAM), have been investigated for hydrogel-based wound dressings. Each material offers distinct advantages but also suffers from limitations. For example, collagen and gelatin provide excellent bioactivity but have poor mechanical stability, while synthetic polymers such as polyvinyl alcohol (PVA) and polyethylene glycol (PEG) offer tunable mechanical properties but lack intrinsic bioactivity and may need functionalization [5]. Among natural polymers, alginate and chitosan are particularly attractive. Alginate (Alg) was selected as the primary matrix due to its biocompatibility, high water-absorption capacity, ability to maintain a moist wound environment, and ease of ionic crosslinking. Chitosan was chosen for its antimicrobial activity, biocompatibility, biodegradability, and structural similarity to glycosaminoglycans in the extracellular matrix [6]. The combination of these two polysaccharides is expected to create a synergistic hydrogel platform combining moisture management, mechanical integrity, and biological activity. Alg is a linear, water-soluble polysaccharide derived from brown algae, composed of repeating units of D-mannuronic acid and L-guluronic acid. It belongs to a group of compounds that are GRAS by the FDA [5]. The HG-forming properties of Alg are determined by the sequence and composition of its monomeric chains. In practice, Alg is most often used in its sodium salt form, which undergoes gelation upon the addition of divalent cations such as calcium and barium, or certain trivalent cations such as iron and aluminum. These cations induce ionic crosslinking between the carboxyl groups of the polymer chains, resulting in the formation of a stable gel network. Alginate offers several key advantages for wound dressing applications, including: (i) high water absorption capacity, which enables effective management of wound exudates; (ii) biocompatibility and non-toxicity, making it suitable for contact with living tissues; (iii) mild and non-toxic gelation conditions, which allow incorporation of bioactive agents without denaturation; (iv) tunable degradation behavior through crosslinking density; and (v) cost-effectiveness and widespread availability [5,6]. Studies have demonstrated that the chemical structure, molecular size, and gelation process of Alg HGs play a critical role in defining their properties, including swelling behavior, stability, biodegradation, safety profile, and biocompatibility [6,7].

In addition to its biocompatibility, this biopolymer exhibits strong cell-adhesive and antimicrobial properties. Due to its structural similarity to natural glycosaminoglycans found in the extracellular matrix of skin, chitosan facilitates the migration of essential biomolecules to the wound site, thereby accelerating the repair process [8,9]. The polycationic nature of chitosan enables electrostatic interactions with negatively charged bacterial cell membranes, contributing to its antimicrobial activity. Recent studies have further demonstrated the potential of chitosan-based nanocomposites for tissue regeneration applications, where the synergistic combination of polymeric matrices, bioactive nanoparticles, and natural compounds has shown enhanced wound-healing efficacy. Furthermore, owing to its polymer chain length and polycationic nature, chitosan (Chi) can cover nerve endings and contribute to pain control. These unique biological and intrinsic properties make Chi a highly promising material for wound healing applications [[10], [11], [12]].

HA nanoparticles exhibit unique properties due to their high surface-to-volume ratio. Among these, the enhanced adhesion of proteins and osteoblasts to these nanoscale ceramic particles has been previously reported [13]. Although hydroxyapatite is widely used in bone-related applications, recent studies have demonstrated its utility in wound-healing materials because of its excellent biocompatibility, ability to reinforce polymeric networks, modulation of swelling and degradation behavior, potential pH-buffering capacity, and promotion of cellular responses [14]. The incorporation of HA nanoparticles into polymeric matrices also leads to a significant improvement in the mechanical properties of the resulting composites [15]. Thymus vulgaris, commonly known as thyme, is one of the oldest medicinal plants and belongs to the Lamiaceae family. This plant contains a wide variety of bioactive compounds, including tannins, flavonoids, saponins, bitter substances, phenolic compounds such as thymol, carvacrol, p-cymene, linalool, cineole, terpenoids, glycosides, caffeic acid, and rosmarinic acid. Thymol, a phenolic compound, is considered the principal active ingredient, while carvacrol represents another major constituent. Both are highly soluble in alcohol and organic solvents and are primarily accumulated in the young leaves during plant growth, where they play a crucial role in the antimicrobial activity of thyme [16,17].

The combination of Alg-Chi HGs with HA nanoparticles and TE can provide a multifunctional and highly efficient system for advanced WDs. Alg, serving as the primary matrix, ensures fluid absorption and the maintenance of a moist wound environment, while Chi, with its inherent antibacterial activity and excellent BC, accelerates tissue regeneration [18,19]. The incorporation of HA nanoparticles not only enhances the mechanical strength of the scaf but also promotes cellular interactions, induces bioactivity, and facilitates tissue remodeling. Furthermore, the addition of TE, rich in phenolic compounds such as thymol and carvacrol, imparts strong antimicrobial activity and effectively prevents wound infection [20]. The synergistic effects of these components can improve the rate of healing, reduce inflammation, and enhance the quality of regenerated tissue, ultimately offering a promising strategy for the development of next-generation WDs with clinical applications [21,22].

In previous studies, the development of such systems has also been investigated to improve the performance of WDs. Straccia et al. [23] developed Alg HGs coated with Chi hydrochloride (CH-Cl) to enhance their performance as WDs. Coated HGs retained high water content (≈65–70 wt%) but showed reduced swelling (from ∼460% in uncoated Alg1 to ∼200% in c-Alg1) and improved stability in saline, with negligible weight loss compared to ∼10% in uncoated samples after 8 h. Drug release tests using rhodamine B revealed that uncoated HGs released 70–80% of the drug within 8 h, whereas coated ones released only 50–60%. Cytotoxicity assays confirmed no acute toxicity of CH-Cl (0.1% w/v) to human mesenchymal stromal cells over 7 days. Antibacterial tests against *Escherichia coli* demonstrated >99% bacterial inactivation within 3 h and complete killing after 24 h for certain coated samples. However, this system exhibited significantly reduced swelling capacity (from ∼460% to ∼200%), which may limit its ability to manage high exudate volumes in chronic wounds. Additionally, it did not incorporate bioactive ceramic reinforcements or natural antimicrobial extracts, leaving mechanical reinforcement and broad-spectrum antimicrobial activity unaddressed. Bagher et al. [24] evaluated Alg/Chi HGs loaded with hesperidin for wound healing. The HGs showed high porosity (∼91%), suitable pore sizes (63-144 μm), good swelling (∼344%), and ∼80% degradation over 14 days. Hesperidin was released gradually (∼77% in 14 days). Hemocompatibility was confirmed, and HGs, especially Alg/Chi/10%Hes, effectively inhibited *S. aureus* and *P. aeruginosa*. In vitro, 3T3 fibroblast proliferation increased with hesperidin, peaking at 10%. In vivo, rat full-thickness wounds treated with Alg/Chi/10%Hes showed 82% closure at day 7 and 98% at day 14, outperforming gauze controls (59%). Histology revealed enhanced epithelialization, collagen deposition, and angiogenesis. Despite these promising results, the mechanical properties of their system were not comprehensively evaluated, and the degradation profile was not correlated with swelling behavior or structural stability. Furthermore, the study did not investigate adsorption kinetics or controlled release mechanisms in detail. Zhang et al. [25] studied a novel sodium Alg/Chi oligosaccharide-zinc oxide (SA-COS-ZnO) composite hydrogel for wound healing applications. The hydrogel was formed through a spontaneous Schiff base reaction between oxidized Alg and Chi oligosaccharide, without requiring chemical crosslinkers, and incorporated ZnO nanoparticles to enhance mechanical strength and antibacterial activity. The resulting hydrogel exhibited favorable properties, including high porosity (80%), swelling capacity (150%), and moisture vapor transmission rate (682 g/m²/24h). It demonstrated excellent biocompatibility with blood and cells, strong broad-spectrum antibacterial effects against pathogens such as *E. coli* and *S. aureus*, and significantly accelerated wound healing in a rat scald model compared to control groups. The study highlights the potential of marine carbohydrate-based HGs as effective WDs.

Saberian et al. [26] evaluated and characterized Alg/Chi-based HGs incorporating honey and Aloe vera for wound dressing applications. Four hydrogel formulations were prepared and analyzed by SEM and FTIR, revealing a porous structure with interconnected cavities ranging from 111 to 162 μm, suitable for cell adhesion, migration, and proliferation. Physical assessments showed that the Al-Chi-Alv hydrogel had the highest porosity (exact value not reported) and a swelling rate comparable to the basic HG, while honey-containing HGs had slightly lower swelling rates. WVTR and contact angle measurements indicated good moisture permeability and hydrophilicity (contact angle 43.3° for Al-Chi-Alv). Antibacterial tests demonstrated inhibitory zones of 23 mm against *Staphylococcus aureus* and 14 mm against *Pseudomonas aeruginosa* for the Al-Chi-Alv-H HG. Biocompatibility and hemolysis tests showed cell viability increased over 7 days and hemolysis rates below 5%. Overall, the combination of honey and Aloe vera produced a biocompatible, porous, and antibacterial hydrogel suitable for wound healing.

Although alginate-, chitosan-, and hydroxyapatite-based biomaterials have been extensively investigated for wound-healing applications, most reported systems focus on only one or two functional aspects, such as swelling behavior, mechanical reinforcement, or antimicrobial activity. For instance, while Straccia et al. [23] successfully demonstrated the antimicrobial and sustained-release properties of alginate-chitosan hydrochloride-coated hydrogels, their system exhibited reduced swelling capacity (from ∼460% to ∼200%) and did not incorporate bioactive ceramic reinforcements or natural antimicrobial extracts to enhance multifunctionality. Similarly, Bagher et al. [24] reported promising wound-healing efficacy for alginate/chitosan hydrogels loaded with hesperidin; however, the mechanical properties of their system were not comprehensively evaluated, and the degradation profile was not correlated with swelling behavior or structural stability. Zhang et al. [25] demonstrated the effectiveness of alginate-chitosan oligosaccharide-ZnO composite hydrogels for wound healing, yet their study did not investigate the adsorption and release kinetics of the incorporated bioactive agents in detail, nor did it evaluate the long-term degradation behavior of the system. Saberian et al. [26] developed alginate/chitosan hydrogels with honey and Aloe vera, achieving promising antibacterial and biocompatible properties; however, the mechanical properties of their hydrogels were not systematically characterized, and the release behavior of the incorporated natural therapeutics was not investigated. Furthermore, studies integrating hydroxyapatite nanoparticles and *Thymus vulgaris* extract within a single hydrogel platform and systematically correlating composition with adsorption behavior, release kinetics, biodegradation, cytocompatibility, and antimicrobial performance remain limited.

Consequently, several unresolved challenges remain, including: (i) the lack of a single system that simultaneously provides mechanical reinforcement, controlled drug release, structural stability, and broad-spectrum antimicrobial activity; (ii) insufficient understanding of how the combination of mineral reinforcements and plant-derived bioactive compounds influences the structure-property relationships of alginate-chitosan hydrogels; and (iii) the absence of comprehensive studies linking material composition with adsorption behavior, release kinetics, biodegradation, and cytocompatibility in a single platform. Therefore, the development of multifunctional wound dressings capable of simultaneously providing structural stability, biological activity, sustained release, and broad-spectrum antimicrobial protection remains an important challenge.

To address existing research gaps, the present study developed an alginate/chitosan hydrogel reinforced with hydroxyapatite nanoparticles and loaded with *Thymus vulgaris* extract. Alginate was selected as the primary hydrogel matrix because of its excellent biocompatibility, high water-absorption capacity, and ability to maintain a moist wound environment. Chitosan was incorporated to introduce additional bioactive amino groups, enhance antimicrobial performance, and improve matrix integrity through polyelectrolyte interactions with alginate. Hydroxyapatite was selected not only as a reinforcing phase but also because of its bioactivity, calcium-ion-mediated interactions, and potential influence on adsorption and release behavior. *Thymus vulgaris* extract was incorporated as a natural source of antioxidant and antimicrobial phytochemicals, particularly phenolic and terpenoid compounds. The novelty of this work lies in the synergistic integration of these components within a single multifunctional hydrogel and the comprehensive evaluation of the resulting structure–property relationships through morphological, structural, mechanical, swelling, loading, release, biodegradation, cytocompatibility, and antimicrobial studies. Unlike previous studies that focused on isolated functional improvements, the present work provides a systematic investigation of how the combined incorporation of HA, Chi, and TE affects the overall performance of the hydrogel system. This comprehensive approach addresses the identified research gaps by offering a balanced optimization of mechanical integrity, swelling behavior, structural stability, controlled release capability, biocompatibility, and antimicrobial activity within a single multifunctional platform. The developed Alg/HA/Chi/TE hydrogel is proposed as a promising platform for advanced wound-dressing applications and sustained delivery of natural therapeutic agents.

### 2.1 Materials

Sodium Alg (CAS No. 9005-38-3, viscosity of 500 cP, molecular weight of 216.12 kDa) and Chi (CAS No. 9012-76-4, viscosity of 200-800 cP, molecular weight of 150 kDa and deacetylation degree of 75-85%) were purchased from Sigma-Aldrich and used without further purification. HA nanoparticles (Ca₁₀(PO₄)₆(OH)₂, <200 nm, 99.8%, molecular weight of 502.31 kDa) with a calcium-to-phosphate molar ratio of 1.67 were obtained from Merck, Germany. Wild Iranian mountain thyme (*Thymus vulgaris*) was procured from a local market for extraction purposes. Double-distilled deionized water was used as the solvent throughout the experiments after ultrasonic treatment to remove any residual chlorine contaminants.

### 2.2 Methods

#### 2.2.1 Thyme extract preparation

The thyme leaves were carefully separated from stems, washed thoroughly with deionized water, and dried at 40 °C for 48 hours in a laboratory oven. The dried leaves were ground into fine powder using a mechanical grinder. The extraction was carried out using a Soxhlet apparatus with absolute ethanol as the solvent. Approximately 20 g of dried thyme powder was placed in the thimble and extracted with 200 mL of ethanol for 6 hours at 70 °C, ensuring complete extraction of bioactive compounds. The extract was then filtered through Whatman No. 1 filter paper and concentrated using a rotary evaporator at 45 °C under reduced pressure. The resulting crude extract was further dried in a vacuum oven at 40 °C for 24 hours to remove any residual solvent. The final dried extract was stored in airtight containers at 4 °C for further use.

#### 2.2.2 Hydrogel fabrication

The composite hydrogels were prepared using the freeze-drying method. A 2% (w/v) sodium Alg solution was prepared by dissolving alginate in deionized water under magnetic stirring at 500 rpm for 4 hours at room temperature until a homogeneous and bubble-free solution was obtained. Simultaneously, 1.5% (w/v) Chi solution was prepared in 1% acetic acid with 24-hour stirring. HA nanoparticles (1% w/w relative to total polymer) were dispersed in Alg solution using probe sonication (200 W, 15 min). The Chi solution was added dropwise to the Alg-HA mixture with continuous stirring at 400 rpm for 2 hours.

For TE incorporation, a stock solution was first prepared by dissolving the dried extract in absolute ethanol at a 50 mg/mL concentration, followed by sonication for 10 minutes at 35 °C. This solution was then added dropwise to the polymer mixture to achieve a final concentration of 0.5% w/w TE relative to total polymer content. The incorporation was performed under continuous mechanical stirring at 300 rpm for 45 minutes at 25 °C, with pH maintained at 6.0-6.5 using 0.1M NaOH/HCl solutions. The process was carried out in amber containers to prevent photodegradation of bioactive compounds.

The prepared hydrogel mixture was poured into Petri dishes, subsequently immersed in 2% (w/v) CaCl₂ solution under gentle stirring (300 rpm) for 30 min to achieve ionic crosslinking. The volume ratio of crosslinking solution to hydrogel samples was maintained at approximately 10:1 (v/w) to ensure sufficient availability of Ca²⁺ ions for homogeneous gel formation. After crosslinking, the samples were washed thoroughly with deionized water to remove excess calcium ions, and freeze-dried (Christ Alpha 1-2 LDplus) at -50 °C under 0.001 mbar for 48 h. The final scaffold was stored at 4 °C in sealed containers until further characterization. Following freeze-drying, the scaffolds were stored at 4 °C in sealed containers. It should be noted that the samples were not subjected to sterilization prior to characterization or biological evaluation, as sterilization validation was beyond the scope of the present study. For all experiments, samples were prepared from three independently fabricated batches (n = 3), with each batch representing a separate preparation cycle. From each batch, at least three individual specimens were tested per measurement to account for within-batch variability. The following formulations were prepared: Alg, HA, Alg/HA, Alg/HA/Chi, and Alg/HA/Chi/Ext. All characterizations were performed on samples from all formulations unless otherwise specified.

#### 2.2.3 Characterization of hydrogels

The surface morphology and microstructure of the scaffolds were examined using field-emission scanning electron microscopy (FESEM, Hitachi SU-70, Japan) at an accelerating voltage of 15 kV. Prior to imaging, samples were mounted on aluminum stubs using conductive carbon tape and sputter-coated with a thin layer of gold to improve conductivity. Quantitative pore size analysis was performed using *ImageJ* software (NIH, USA) based on FESEM micrographs. Pore diameters were measured from randomly selected regions of representative images to obtain statistically meaningful morphological information. At least 50 pores were measured per sample. However, FESEM imaging was performed on one representative sample per formulation. Standard deviation values based on multiple independently prepared samples could not be calculated, which should be addressed in future studies.

Fourier transform infrared (FTIR) spectroscopy (PerkinElmer Spectrum Two, USA) was used to investigate the chemical structure and intermolecular interactions within the hydrogel formulations. Dried samples were analyzed in the spectral range of 4000–400 cm⁻¹ with a resolution of 4 cm⁻¹ and 32 accumulated scans per spectrum.

The crystalline characteristics of the samples were evaluated using X-ray diffraction (XRD, Philips X'Pert Pro, Netherlands) equipped with Cu Kα radiation (λ = 1.5406 Å). Diffraction patterns were recorded over a 2θ range of 5–80° at room temperature with a scanning rate of 2° min⁻¹.

Mechanical properties were determined using a universal testing machine (Zwick/Roell Z050, Germany) equipped with a 50 N load cell. Rectangular specimens of known dimensions were subjected to uniaxial tensile testing at a crosshead speed of 5 mm min⁻¹ until failure. Tensile strength, elongation at break, and toughness were calculated from the resulting stress–strain curves. At least three specimens were tested for each formulation, and the results are reported as mean ± standard deviation. Sample dimensions were measured prior to testing and used for stress calculations.

The swelling behavior of the hydrogel scaffolds was evaluated by gravimetry in phosphate-buffered saline (PBS, pH 7.4) at 37 °C. Pre-weighed dry samples (W₀) were immersed in PBS and removed at predetermined time intervals. Excess surface liquid was carefully removed using filter paper, and the swollen weight (Wt) was recorded. The swelling ratio was calculated according to Eq. (1):

(1) $$\text{Swelling}\left(\%\right)=\left(\left(\text{Wt}-W_{0}\right)/W_{0}\right)\times 100$$

#### 2.2.4 Cell viability assessment (MTT assay)

Cell viability was evaluated using the MTT assay according to ISO 10993-5 guidelines. L929 mouse fibroblast cells were cultured in Dulbecco’s Modified Eagle Medium (DMEM) supplemented with 10% fetal bovine serum and 1% penicillin–streptomycin under standard incubation conditions (37 °C, 5% CO₂).

Hydrogel extracts were prepared by incubating sterilized samples in complete culture medium under standard conditions. The obtained extracts were subsequently applied to L929 cells and incubated for 24, 48, and 72 h. At each time point, MTT solution was added and incubated to allow metabolically active cells to reduce MTT into insoluble formazan crystals. The crystals were dissolved using an appropriate solvent, and absorbance was measured at 570 nm using a microplate reader. Cell viability was calculated as a percentage relative to untreated control cells according to Eq. (2):

(2) $$\text{Cell}\;\text{Viability}\;(\%)=\frac{A_{sample}}{A_{control}}\times 100$$

where $A_{sample}$ and $A_{control}$ represent the absorbance values of treated and untreated cells, respectively.

#### 2.2.5 Antimicrobial evaluation

##### 2.2.5.1 Microbial strains and culture conditions

Antimicrobial activity was evaluated against the Gram-positive bacterium *Bacillus cereus* (ATCC 11778), the Gram-negative bacterium *Agrobacterium tumefaciens* (ATCC 23380), and the fungal strains *Aspergillus flavus* (ATCC 9643), *Fusarium avenaceum* (ATCC 58712), and *Fusarium solani* (ATCC 36031). These microorganisms were selected as representative Gram-positive, Gram-negative, and fungal species commonly employed in antimicrobial biomaterial studies to evaluate broad-spectrum antimicrobial performance.

Bacterial strains were cultured in Mueller–Hinton broth at 37 °C for 24 h, whereas fungal strains were maintained on Sabouraud dextrose agar at 28 °C for 48–72 h. The turbidity of bacterial suspensions was adjusted to 0.5 McFarland standard (approximately 1.5 × 10⁸ CFU/mL).

##### 2.2.5.2 Growth inhibition assay

Antimicrobial activity was quantified using the growth-inhibition method. Hydrogel extracts were prepared by incubating 1 cm² samples in 5 mL sterile distilled water for 24 h at 37 °C. For bacterial assays, 100 μL of microbial suspension was mixed with 100 μL of hydrogel extract in 96-well microplates and incubated at 37 °C for 24 h. For fungal assays, spore suspensions (1 × 10⁶ spores/mL) were used under the same procedure.

The optical density (OD) was measured at 600 nm using a microplate reader, and the inhibition percentage was calculated according to Eq. (3):

(3) $$\text{Inhibition}\;(\%)=\left(1\frac{OD_{sample}}{OD_{control}}\right)\times 100\;$$

Ciprofloxacin (10 μg/mL) and amphotericin B (20 μg/mL) were used as positive controls for antibacterial and antifungal experiments, respectively. In addition, unloaded formulations (Alg, Alg/HA, and Alg/HA/Chi) were evaluated as negative controls to distinguish the intrinsic antimicrobial activity of the matrix components from the additional effect of thyme extract.

##### 2.2.5.3 Minimum inhibitory concentration (MIC)

The MIC values were determined using the broth microdilution method according to CLSI guidelines (M07-A9 and M38-A2). Serial two-fold dilutions of hydrogel extract solutions were prepared in appropriate broth media. The MIC was defined as the lowest concentration that completely inhibited visible microbial growth after incubation at 37 °C for 24 h for bacteria and at 28 °C for 48 h for fungi.

#### 2.2.6 Thyme extract release study

The release behavior of thyme extract (TE) from the hydrogel matrices was investigated in PBS (pH 7.4) at 37 °C under gentle shaking conditions. At predetermined time intervals, aliquots of the release medium were withdrawn and replaced with equal volumes of fresh PBS to maintain sink conditions. The concentration of released thymol, selected as the principal bioactive marker compound of TE, was quantified using a UV–Vis spectrophotometer by measuring absorbance at 274 nm. The cumulative release percentage was calculated relative to the initially loaded amount of TE.

#### 2.2.7 Statistical analysis

All experiments were performed in triplicate (n=3), where each replicate represents an independently prepared sample from a separate fabrication batch. Results were expressed as mean ± standard deviation (SD). One-way analysis of variance (ANOVA) followed by Tukey's post-hoc test was used for statistical comparisons, with p < 0.05 considered statistically significant. The factor (independent variable) in all ANOVA analyses was the hydrogel formulation (five levels: Alg, HA, Alg/HA, Alg/HA/Chi, and Alg/HA/Chi/Ext). The responses (dependent variables) included: (i) mechanical properties (UTS, Young's modulus, maximum strain, and toughness), (ii) swelling ratio, (iii) cell viability (at 24, 48, and 72 h), (iv) extract loading capacity, (v) cumulative release percentage, (vi) biodegradation weight loss, (vii) stability weight loss, and (viii) antimicrobial inhibition percentage and MIC values. Prior to ANOVA, the data were tested for normality using the Shapiro-Wilk test and for homogeneity of variances using Levene's test. All data met the assumptions of normality (p > 0.05) and homogeneity of variances (p > 0.05), so no data transformation or normalization was required. The symbols used in figures denote: *P < 0.001 vs. Alg; ###P < 0.001 vs. Alg/HA; $P < 0.05 vs. Alg/HA/Chi.

## 3 Results

### 3.1 Microstructural analysis

**Fig. 1:**
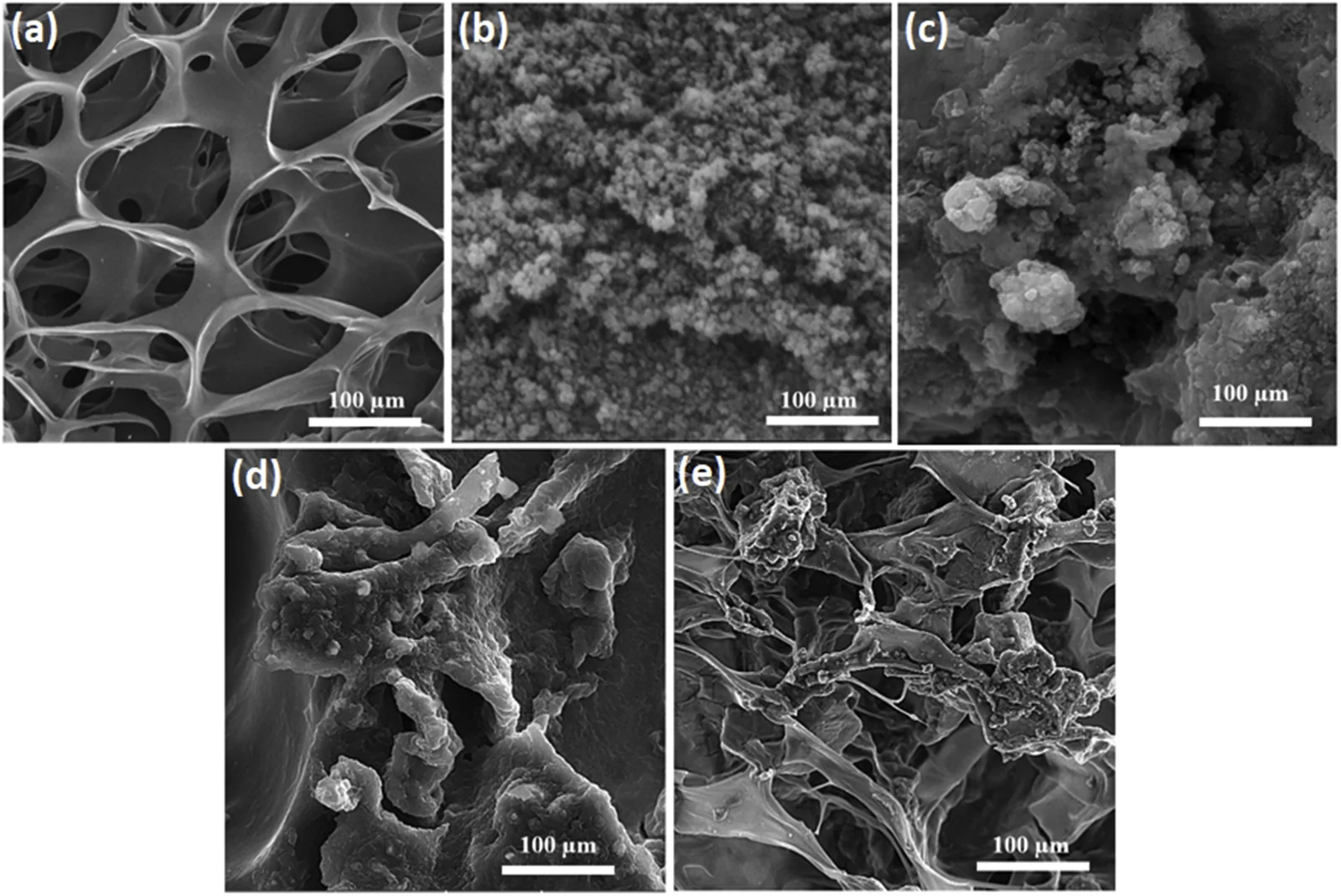
FE-SEM micrographs of (a) Alg, (b) HA, (c) Alg/HA, (d) Alg/Chi/HA, and (e) Alg/HA/Chi/extract samples.

FE-SEM information is essential for understanding the mechanical behavior, BC, fluid exchange capability, and drug release potential of HG-based wound dressing systems [27,28]. Therefore, FESEM analysis is a decisive factor in evaluating the success of the design and fabrication of these materials. Fig. 1 presents FESEM micrographs of Alg, HA, Alg/HA, Alg/Chi/HA, and Alg/HA/Chi/extract samples. In Fig. 1a, corresponding to pure Alg hydrogel, a porous structure with relatively large and irregular pores in the range of 20–150 μm is observed. The thin walls between these pores indicate weak connectivity among the polymer chains, leading to an open network with abundant void spaces. The absence of well-defined crystalline features confirms that Alg remains in an amorphous state. Fig. 1b illustrates the pure HA sample, consisting of fine particles with distinct boundaries and a relatively rough and heterogeneous surface. The particles appear quasi-spherical with an average size of about 0.5–1 μm, reflecting the crystalline nature of this mineral phase. In Fig. 1c, the Alg/HA composite shows mineral particles distributed in agglomerated clusters within the polymer matrix, which alters the porous morphology of the hydrogel. Compared to pure Alg, reduced porosity is observed, suggesting that mineral particles partially fill the voids, leading to relative densification. Fig. 1d, corresponding to the Alg/HA/Chi sample, reveals a more porous and uniform structure compared to the Alg/HA composite, although particle agglomeration remains evident. Smaller and more regular pores are observed at the surface. Fig. 1e, representing the Alg/HA/Chi/Ext sample, shows a structure with increased porosity compared to earlier samples (except pure Alg). The pores display irregular morphology, with some regions partially blocked. Localized aggregations or soft patches are observed in some areas. Overall, the FESEM micrographs demonstrate a substantial reduction in porosity upon the addition of HA to Alg, followed by a progressive increase in porosity with the incorporation of Chi and TE. The micrographs suggest the presence of an open porous network with interconnected channels rather than isolated closed pores. However, it should be noted that pore size analysis was performed on representative FESEM images from one sample per formulation, and standard deviation values based on multiple independently prepared samples could not be calculated. This represents a limitation of the present study, and future work should include quantitative pore analysis from multiple independent samples to better establish process reproducibility.

### 3.2 Chemical composition characterizations

#### 3.2.1 FTIR analysis

**Fig. 2:**
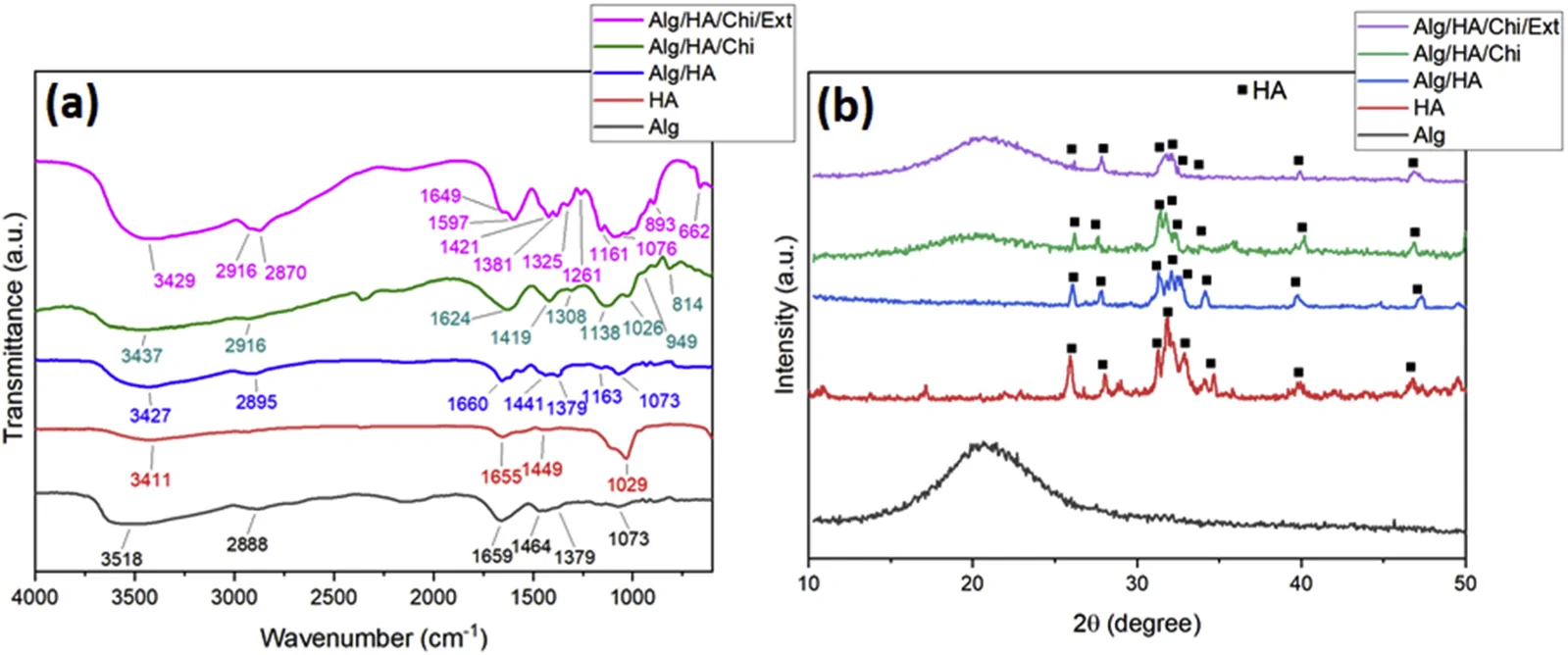
Characterization analyses: (a) FTIR spectra and (b) XRD patterns of Alg, HA, Alg/HA, Alg/Chi/HA, and Alg/HA/Chi/extract samples.

In this section, the significance of FTIR lies in its ability to confirm physical or chemical interactions among the composite constituents, to detect structural changes resulting from the combination of different biomaterials and to monitor the stabilization or formation of new bonds within the system under investigation. Fig. 2(a) presents the FTIR spectra of the studied samples.

The FTIR spectrum of Alg displays a broad absorption band at 3518 cm⁻¹ (O–H stretching), a band near 2888 cm⁻¹ (C–H stretching), bands at 1659, 1464, and 1379 cm⁻¹ (asymmetric stretching of –COO⁻, C=O stretching, and C–H bending), and absorption at 1073 cm⁻¹ (C–O stretching). The spectrum of pure HA shows a broad band at 3411 cm⁻¹ (–OH stretching), a strong absorption at 1655 cm⁻¹ (adsorbed water), and bands at 1449 and 1029 cm⁻¹ (phosphate PO₄³⁻ vibrations). For the Alg/HA composite, characteristic bands of both components are retained. OH and C–H bands are observed at 3427 and 2895 cm⁻¹, with slight shifts and reduced intensities. In the lower wavenumber region, C–O, C–H, and PO₄³⁻ bands at 1073, 1163, 1379, 1441, and 1660 cm⁻¹ are preserved. Upon incorporation of Chi (Alg/HA/Chi), the OH stretching band shifted to 3437 cm⁻¹ with increased intensity. A band appeared at 1624 cm⁻¹ in the amide and amino region. Bands at 1419, 1308, 1138, 1026, and 949 cm⁻¹ are also observed. In the final composite containing TE (Alg/HA/Chi/Ext), the OH/NH stretching band shifts to 3429 cm⁻¹, with new peaks appearing at 2916 and 2870 cm⁻¹. In the 1650–1600 cm⁻¹ region, two distinct peaks are observed at 1649 cm⁻¹ and 1597 cm⁻¹. Additional bands at 1421, 1381, 1325, 1261, 1161, 1076, 893, and 662 cm⁻¹ are present.

#### 3.2.2 XRD analysis

Fig. 2(b) presents the XRD patterns of the investigated samples. Pure Alg exhibits a broad hump around 20°, confirming its amorphous nature. Pure HA displays sharp diffraction peaks at approximately 25.9°, 28.0°, 31.3°, 31.9°, 32.8°, 34.6°, 39.9°, and 46.8°, corresponding to the (002), (210), (211), (112), (300), (202), (310), and (222) crystallographic planes. Upon incorporation of HA into Alg (Alg/HA), the characteristic HA peaks remain visible but with reduced intensities. In the Alg/HA/Chi composite, peak intensities and sharpness decrease further, with peak broadening observed. For the Alg/HA/Chi/Ext sample, further reduction in peak intensity and sharpness is observed, accompanied by a stronger amorphous background. The average crystallite size (D) was estimated using the Scherrer equation. For pure HA, the crystallite size was 42.9 nm. In the Alg/HA, Alg/HA/Chi, and Alg/HA/Chi/Ext samples, the crystallite size decreased progressively to 35.5 nm, 27.8 nm, and 18.8 nm, respectively. The degree of crystallinity (Xc) was estimated as 8% for pure Alg, 89.5% for pure HA, 53.7% for Alg/HA, 33.5% for Alg/HA/Chi, and 21.7% for Alg/HA/Chi/Ext.

### 3.3 Mechanical properties of the composites

The tensile test is one of the most important mechanical characterization methods for assessing the performance and durability of polymeric and bio-based materials, including HGs. The stress–strain curves obtained from tensile tests are presented in Figure S1, and the corresponding mechanical parameters are summarized in Table S1.

As shown in Figure S1 and Table S1, the pure Alg hydrogel exhibited relatively good mechanical properties, attributed to its highly porous structure. Pure Alg exhibited UTS of 25.6 ± 5.5 MPa, Young's modulus of 16.2 ± 0.95 MPa, maximum strain of 2.2 ± 0.27%, and toughness of 291.5 kJ/m³. The Alg/HA sample showed a significant reduction in UTS (11.88 ± 1.45 MPa), modulus (11.7 ± 0.8 MPa), strain (1.27 ± 0.17%), and toughness (75.43 kJ/m³). The Alg/HA/Chi sample showed improved mechanical parameters compared to Alg/HA, with UTS of 20.86 ± 2.2 MPa, modulus of 12.5 ± 1 MPa, strain of 1.96 ± 0.12%, and toughness of 204 kJ/m³. The Alg/HA/Chi/Ext sample showed the highest mechanical performance, with UTS of 50 ± 2.3 MPa, modulus of 16 ± 1.5 MPa, strain of 4.35 ± 0.55%, and toughness of 1087.5 kJ/m³.

### 3.4 Swelling and MTT analyses

#### 3.4.1 Swelling behavior

The swelling results for the studied samples are presented in Figure S2. Pure Alg exhibited the highest swelling ratio of 832% ± 55%. Pure HA exhibited the lowest swelling ratio of 63% ± 13%. The Alg/HA composite showed a swelling ratio of 366% ± 47%. The Alg/HA/Chi sample showed a swelling ratio of 533% ± 49%. The Alg/HA/Chi/Ext sample showed a swelling ratio of 766% ± 54%.

#### 3.4.2 Cell viability (MTT assay)

The MTT results for four hydrogel samples (pure Alg, Alg/HA, Alg/HA/Chi, and Alg/HA/Chi/Ext) measured at 24, 48, and 72 h are presented in Figure S3. At 24 h: Alg showed ∼55% viability, Alg/HA ∼60%, Alg/HA/Chi ∼67%, and Alg/HA/Chi/Ext ∼70%. At 48 h: Alg reached ∼57%, Alg/HA ∼66%, Alg/HA/Chi ∼74%, and Alg/HA/Chi/Ext exceeded 80%. At 72 h: Alg remained ∼59%, Alg/HA ∼70%, Alg/HA/Chi ∼78%, and Alg/HA/Chi/Ext ∼88%.

### 3.5 Loading of TE and analysis of adsorption isotherms

**Fig. 3:**
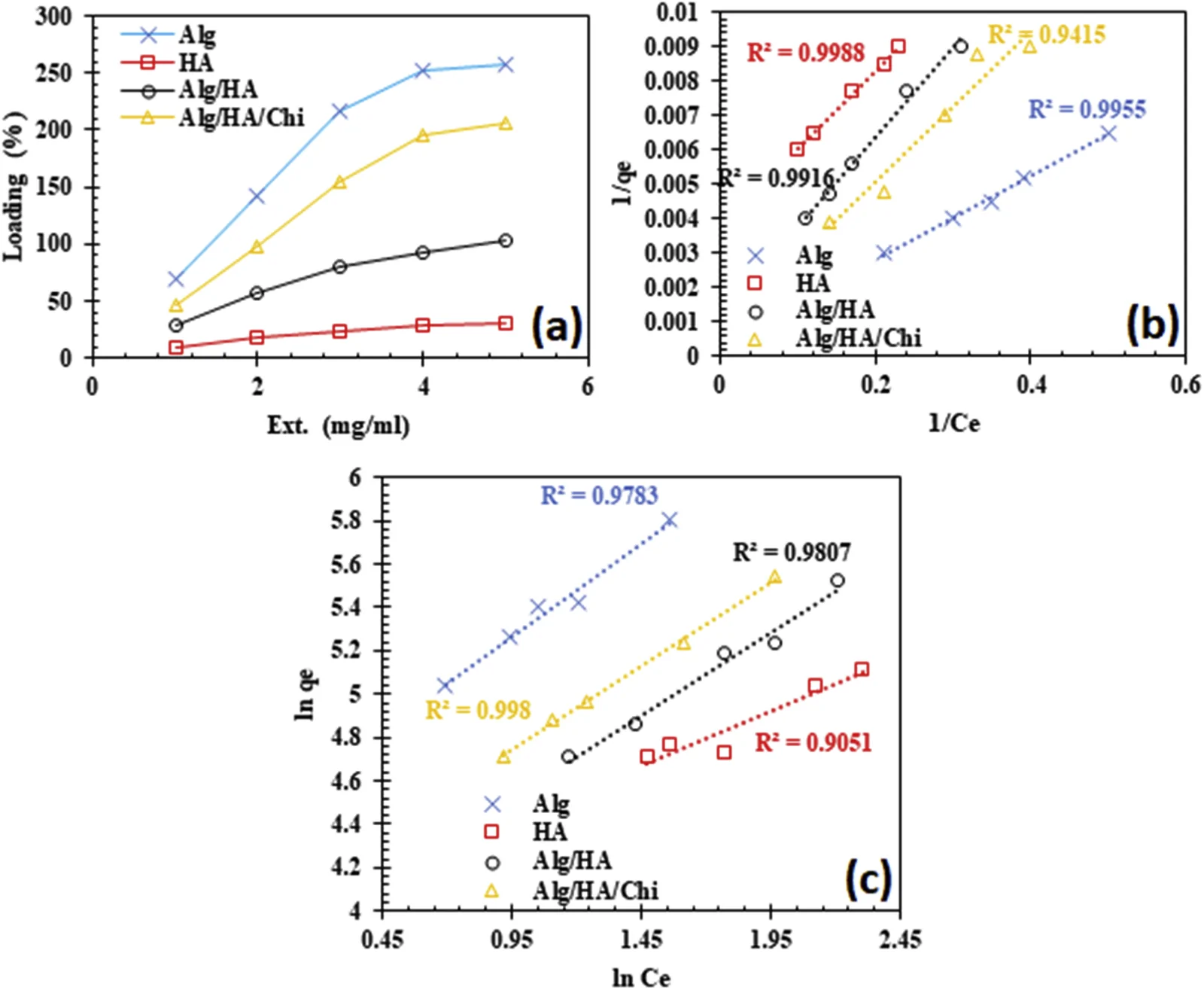
(a) The variations in extract loading percentage and adsorption isotherm plots of (b) Langmuir and (c) Freundlich models for extract adsorption onto Alg, HA, Alg/HA, and Alg/HA/Chi composites.

In this study, the loading of TE onto four different systems (pure Alg, pure HA, the binary Alg/HA composite, and the ternary Alg/HA/Chi composite) was investigated at various extract concentrations ranging from 1 to 5 mg/mL. The corresponding loading efficiencies are presented in Fig. 3a. Pure Alg exhibited the highest loading capacity at all concentrations. Pure HA showed the lowest loading capacity, with a maximum of ∼30%. The Alg/HA and Alg/HA/Chi composites demonstrated intermediate performance. Experimental data were analyzed using Langmuir and Freundlich models (Fig. 3b and 3c), and the derived parameters are summarized in Table S2. The Langmuir maximum adsorption capacity (Q₀) was highest for Alg (2521.15 mg/g), lowest for HA (263.12 mg/g), and intermediate for Alg/HA (833.33 mg/g) and Alg/HA/Chi (1428.75 mg/g). The Langmuir constant (KL) was highest for Alg (0.32), followed by Alg/HA/Chi (0.29), and lowest for HA (0.02). In Freundlich analysis, the relative adsorption capacity (KF) followed the order Alg > Alg/HA/Chi > Alg/HA > HA. The adsorption intensity index (n) exceeded 1 in all cases, with the highest value for Alg (n = 1.99) and the lowest for HA (n = 1.15).

### 3.6 Kinetics of TE release

The cumulative release percentages for each matrix are presented in Figure S4(a). Pure Alg exhibited a relatively uniform and slow release pattern, reaching approximately 30% release after 30 days. Pure HA displayed a burst release profile, with about 59% release within the first seven days, reaching ∼73% by day 30. The Alg/HA composite showed an intermediate release pattern, reaching ∼57% after 30 days. The Alg/HA/Chi system displayed a biphasic, two-step release profile, reaching ∼86% at day 30 Curve fitting revealed that the Korsmeyer–Peppas model provided the best fit with the highest R² values for all formulations (Figure S4(b-f)).

### 3.7 Biodegradation assessment

To evaluate the biodegradation of the prepared systems, Alg, Alg/HA, and Alg/HA/Chi samples were subjected to a natural soil burial test. Soil burial testing is widely used as a preliminary method for comparing the relative biodegradability of biomaterials under natural environmental conditions. In the present study, this test was employed to evaluate the influence of HA and chitosan incorporation on the degradation behavior and structural stability of the prepared hydrogel systems. The weight loss of Alg, Alg/HA, and Alg/HA/Chi samples over 30 days in soil burial test is illustrated in Figure S5. Pure Alg exhibited the highest degradation, reaching approximately 38% weight loss after 30 days. The Alg/HA system showed the lowest degradation rate (around 24%). The Alg/HA/Chi system displayed intermediate behavior, with a weight loss of about 27% after 30 days. All three systems exhibited a two-stage degradation profile.

### 3.8 Hydrogel stability analysis

The stability test conducted in distilled water provided valuable insights into the physicochemical behavior and structural integrity of the different hydrogel systems under aqueous conditions. As shown in Figure S6, the weight loss of the samples was monitored over a period of 72 hours in contact with distilled water. Pure Alg exhibited rapid weight loss during the first 24 hours, reaching approximately 3.5% and remaining nearly constant thereafter. Alg/HA and Alg/HA/Chi samples exhibited much lower and more stable weight loss profiles, with modest initial decreases (approximately 0.8% and 0.92%, respectively) followed by stabilization.

### 3.9 Antibacterial and antifungal evaluation

To assess the antimicrobial performance of the developed bio-composite systems, antibacterial and antifungal activity tests were conducted using growth inhibition percentage and MIC assays. The tests were performed against two bacterial strains (*Bacillus cereus* and *Agrobacterium tumefaciens*) and three fungal species (*Aspergillus flavus, Fusarium avenaceum*, and *Fusarium solani*). The obtained results are illustrated in Figure S7. Pure Alg exhibited inhibition percentages of 45.44% for *B. cereus* and 25.98% for *A. tumefaciens*. Alg/HA showed inhibition percentages of 68.22% and 70.32% for *B. cereus* and *A. tumefaciens*, respectively. Alg/HA/Chi exhibited comparable antibacterial efficiency. Alg/HA/Chi/Ext demonstrated the highest antibacterial activity, achieving 98.99% inhibition against *B. cereus* and over 74% inhibition against *A. tumefaciens*. For *B. cereus*, the MIC decreased from 62.5 µg/mL (Alg) to 3.9 µg/mL (Alg/HA/Chi/Ext). For *A. tumefaciens*, the MIC decreased from 250 µg/mL to 3.66 µg/mL. Pure Alg displayed inhibition ranging from 26.84% to 50.12%. Alg/HA/Chi/Ext showed inhibition rates of 99.12% (*A. flavus*), 56.34% (*F. avenaceum*), and 91.22% (*F. solani*). MIC values for this formulation were below 5 µg/mL for all tested fungi, with 0.59 µg/mL for *A. flavus*.

## 4 Discussion

### 4.1 Microstructural analysis

The FESEM results provide important insights into the structural evolution of the composite hydrogels. The porous structure of pure Alg with thin walls and large pores (20–150 μm) is characteristic of freeze-dried alginate networks and is consistent with previous reports [18,29]. This open network structure, while beneficial for fluid and gas exchange, explains the poor mechanical resistance observed in tensile tests. The pure HA sample exhibited a crystalline, quasi-spherical morphology with particle sizes of 0.5–1 μm, consistent with previous studies [28,30]. The ordered crystalline structure and sharp boundaries reflect the high purity of the mineral phase. In the Alg/HA composite, mineral particles formed agglomerated clusters within the polymer matrix, altering the porous morphology. The reduced porosity compared to pure Alg suggests that HA particles partially fill void spaces, leading to relative densification. This limited compatibility between HA and Alg phases hinders uniform dispersion and explains the reduced mechanical properties observed in tensile testing. The Alg/HA/Chi sample showed improved structural uniformity with smaller and more regular pores. The film-forming ability of chitosan filled micro-gaps within the network, enhancing dispersion of the mineral phase [31]. This structural improvement correlates with the enhanced mechanical properties observed for this formulation. The Alg/HA/Chi/Ext sample exhibited increased porosity with irregular pore morphology. The incorporation of thyme extract appears to enhance distribution of the mineral phase within the polymeric matrix. Localized soft patches likely correspond to phytochemical constituents of the extract, which may facilitate controlled release of bioactive agents. This morphology is advantageous for drug delivery applications. The open porous network with interconnected channels observed in all samples facilitates fluid transport, nutrient diffusion, and cellular infiltration—all critical for wound dressing applications. The combined use of Chi and TE results in a more porous, interconnected, and stable network, supporting their potential for clinical applications in chronic or infected wounds.

### 4.2 Chemical composition characterizations

#### 4.2.1 FTIR analysis

The FTIR results confirm successful incorporation of all components and reveal the nature of intermolecular interactions within the composite system. The characteristic bands of Alg (3518 cm⁻¹, 2888 cm⁻¹, 1659 cm⁻¹, 1464 cm⁻¹, 1379 cm⁻¹, 1073 cm⁻¹) are consistent with previous reports [[32], [33], [34], [35], [36], [37], [38]]. The spectrum of pure HA with bands at 3411 cm⁻¹, 1655 cm⁻¹, 1449 cm⁻¹, and 1029 cm⁻¹ corresponds to hydroxyapatite's characteristic vibrations [35,36]. In the Alg/HA composite, the retention of characteristic bands from both components with slight shifts suggests physical interactions between calcium ions of HA and carboxylate groups of Alg. This interaction explains the reduced swelling and altered mechanical properties observed. The Alg/HA/Chi composite showed more pronounced spectral changes. The shift of the OH stretching band to 3437 cm⁻¹ with increased intensity reflects the contribution of hydroxyl and amino groups from chitosan and formation of an extended hydrogen-bonding network [39,40]. The appearance of the band at 1624 cm⁻¹ and the shift of the carboxylate-related band from 1659 cm⁻¹ to 1624 cm⁻¹ suggest electrostatic interactions between protonated amino groups of chitosan and carboxylate groups of alginate—characteristic of polyelectrolyte complex formation [41,42]. These interactions explain the improved mechanical properties and structural stability of the Alg/HA/Chi system. In the final Alg/HA/Chi/Ext composite, additional spectral changes confirm successful incorporation of thyme extract. The peaks at 2916 and 2870 cm⁻¹ are associated with alkyl or phenolic groups [43]. The band at 1597 cm⁻¹, attributed to C=C stretching in aromatic phenolic rings, confirms the presence of bioactive compounds such as thymol and carvacrol. The reduction or modification of certain Alg- and HA-related bands suggests physical and chemical interactions between the extract and the base matrix. It should be noted that the primary crosslinking mechanism is the ionic interaction between Ca²⁺ ions and alginate carboxylate groups (egg-box structure). HA particles, chitosan chains, and TE molecules are incorporated through hydrogen bonding, electrostatic interactions, and physical entrapment rather than direct crosslinking. This hierarchical network structure explains the synergistic improvement in multiple properties.

#### 4.2.2 XRD analysis

The XRD results confirm the presence and stability of the crystalline HA phase within the composites and demonstrate how component incorporation affects crystallinity. The broad hump around 20° for pure Alg confirms its amorphous nature [44,45]. Pure HA displays sharp diffraction peaks matching JCPDS card No. 09-0432, confirming its highly ordered hexagonal structure [46,47]. The reduced peak intensities in Alg/HA compared to pure HA suggest uniform dispersion of HA nanoparticles and structural interference between amorphous Alg and crystalline HA phases [19]. This dispersion, while desirable for homogeneity, explains why HA alone did not provide expected reinforcement—the crystalline order was disrupted. In Alg/HA/Chi, further reduction in peak intensities and broadening indicate reduced crystallite size and increased amorphous fraction due to interactions among HA, Chi, and Alg. The weak diffraction signal of semi-crystalline Chi near 20° overlaps with the amorphous halo of Alg. The most pronounced structural modifications occur in Alg/HA/Chi/Ext. The further reduction in peak intensity and sharpness with stronger amorphous background highlights the inhibitory role of phytochemical compounds in TE on HA crystal growth. Polyphenols, terpenes, and flavonoids form hydrogen bonds or surface complexes with calcium and phosphate ions, hindering HA crystallization [48]. This yields a less crystalline structure with smaller crystallites (18.8 nm vs. 42.9 nm for pure HA). The progressive decrease in crystallinity from 89.5% (pure HA) to 21.7% (Alg/HA/Chi/Ext) demonstrates how increasing structural complexity and component interactions reduce long-range order. While this reduction in crystallinity may be considered a loss of HA's inherent structural character, it creates hybrid materials with potentially enhanced biological performance due to increased surface area and amorphous character.

### 4.3 Mechanical properties

The tensile test results reveal important structure–property relationships in the composite hydrogels. Pure Alg exhibited relatively good mechanical properties (UTS: 25.6 ± 5.5 MPa, toughness: 291.5 kJ/m³), consistent with previous findings [49]. This reflects a balance between stiffness and ductility arising from its amorphous and open structure [34,50]. The significant reduction in all mechanical parameters for Alg/HA (UTS: 11.88 ± 1.45 MPa, toughness: 75.43 kJ/m³) is noteworthy. Although HA is frequently employed as a reinforcing phase in polymeric biomaterials, reinforcement is strongly dependent on particle dispersion, interfacial adhesion, and preservation of polymer network integrity. In this case, the non-uniform dispersion of HA particles created stress concentration sites and premature failure. The denser structure and reduced porosity observed in FESEM increased brittleness and limited deformation capacity. The partial disruption of the continuous alginate network and reduction in effective ionic crosslink density prevented realization of HA's reinforcing potential in the binary system.The marked improvement in mechanical parameters upon Chi addition (Alg/HA/Chi: UTS 20.86 ± 2.2 MPa, toughness 204 kJ/m³) can be attributed to formation of a polyelectrolyte network through electrostatic interactions between chitosan amino groups and alginate carboxylate groups. These interactions enhanced interfacial adhesion, reduced structural defects, and promoted more homogeneous stress distribution. The film-forming ability of chitosan helped fill voids and increased structural continuity. The remarkable improvement in the Alg/HA/Chi/Ext sample (UTS: 50 ± 2.3 MPa, toughness: 1087.5 kJ/m³) demonstrates synergistic reinforcement. The TS nearly doubled relative to pure Alg, while toughness increased nearly fourfold. This behavior suggests that phytochemical constituents of TE (particularly phenolic and flavonoid compounds) interact with the alginate–chitosan network through hydrogen bonding and secondary intermolecular interactions. These interactions strengthen interchain associations while maintaining sufficient chain mobility, enabling efficient stress transfer and delaying crack propagation.

For comparison, superstrong alginate hydrogels prepared via anisotropic densification have shown tensile strengths of 8–57 MPa [51], while alginate filaments from concentrated solutions have reached 36.6–161.3 MPa [52]. Conventional alginate-chitosan hydrogels typically exhibit much lower tensile strengths of 0.55–16.4 MPa [53]. The toughness of our optimized hydrogel (137.6 MJ/m³) substantially exceeds that of biaxially constrained SA/PAM hydrogels (10.20 MJ/m³) [54] and dynamic nanocomposite alginate hydrogels (89.54 kJ/m³) [55]. The simultaneous increase in tensile strength, elongation, and toughness indicates formation of a resilient network architecture capable of dissipating mechanical energy effectively—highly desirable for wound dressing materials subjected to repeated deformation.

### 4.4 Swelling and MTT analyses

#### 4.4.1 Swelling behavior

The swelling results correlate well with structural observations. Pure Alg exhibited the highest swelling (832% ± 55%) due to its highly porous, thin-walled, amorphous network [56,57]. While beneficial for exudate management, this high swelling compromises mechanical strength. The reduced swelling in Alg/HA (366% ± 47%) reflects pore blockage and reduced permeability caused by mineral particles. Additionally, interactions between HA particles and polymer chains act as physical crosslinking sites, restricting chain mobility and reducing free volume for water diffusion. This explains the improved dimensional stability but also the compromised mechanical properties. The increased swelling in Alg/HA/Chi (533% ± 49%) arises from hydrophilic amino and hydroxyl groups in chitosan [58,59], which increase matrix affinity for water. Electrostatic interactions between chitosan and alginate promote formation of a homogeneous polyelectrolyte network that, combined with improved HA dispersion, creates a porous but structurally stable architecture. The high swelling of Alg/HA/Chi/Ext (766% ± 54%), close to pure Alg, demonstrates that TE phytochemicals (flavonoids, polyphenols, terpenes) interact with polymer chains through hydrogen bonding, forming a flexible and interconnected network while preserving free volume for water penetration [60,61]. This balance between hydration capacity and structural integrity is particularly advantageous for wound dressings requiring efficient exudate management and maintenance of a moist healing environment. Overall, swelling behavior is governed by the balance between network density, pore architecture, and polymer–filler interactions established during calcium-mediated crosslinking. The observed trends demonstrate that engineering crosslinking density and network architecture enables simultaneous optimization of swelling and mechanical properties.

#### 4.4.2 Cell viability

The progressive increase in cell viability from Alg to Alg/HA/Chi/Ext highlights the synergistic contribution of each component. The relatively low viability of pure Alg at 24 h (∼55%) may be attributed to its limited cell-adhesive properties rather than cytotoxicity. Although alginate is biocompatible, it lacks specific bioactive motifs for cell attachment, resulting in reduced initial adhesion and metabolic activity [62]. The gradual increase over time confirms non-toxicity. HA incorporation increased viability to ∼60% due to its bioactivity and chemical similarity to bone mineral [63]. Chitosan further enhanced viability to ∼67% through protonated amino groups that interact electrostatically with cell membranes, improving adhesion [8,64,65]. The Alg/HA/Chi/Ext sample exhibited the highest viability (∼70% at 24 h, ∼88% at 72 h), indicating that phytochemicals such as thymol and polyphenols provide antioxidant and anti-inflammatory effects that create a more favorable cellular microenvironment [66,67]. The progressive increase from 24 to 72 h suggests cumulative effects of bioactive components over time. The significantly higher viability at all time points for TE-containing formulations confirms that the extract enhances cytocompatibility. Beyond their contributions to mechanical and biological activity, HA and Chi may also provide hemostatic benefits. Chitosan promotes platelet adhesion and erythrocyte aggregation through electrostatic interactions, while HA provides bioactive surfaces for protein adsorption and platelet activation. Although coagulation was not directly investigated, these properties suggest multifunctionality for wound dressing applications.

### 4.5 Loading of TE and adsorption isotherms

The loading results demonstrate that extract loading is governed by the interplay between surface chemistry, network architecture, and adsorption-site heterogeneity. Pure Alg's highest loading capacity at all concentrations reflects its highly porous network, swelling capacity, and abundant carboxylate groups for electrostatic and hydrogen-bonding interactions with extract compounds [18]. Pure HA's lowest loading capacity (∼30%) is consistent with its low swelling ratio and limited adsorption sites. The rigid crystalline structure and lower surface accessibility restrict extract uptake. The intermediate performance of Alg/HA and Alg/HA/Chi reflects the coexistence of polymeric adsorption domains and mineral surfaces. In Alg/HA, HA particles partially occupied the porous alginate network, reducing accessible adsorption sites. In Alg/HA/Chi, amino and hydroxyl groups from chitosan introduced additional binding sites, enhancing surface heterogeneity and binding-site diversity. The adsorption isotherm analysis reveals important mechanistic insights. Strong agreement of Alg, HA, and Alg/HA data with the Langmuir model indicates monolayer adsorption on relatively uniform binding sites. The particularly good Langmuir fit for HA suggests a more homogeneous surface with limited energetically similar sites. The Langmuir model (Eq. 4) assumes uniform monolayer adsorption on finite, energetically equivalent sites, whereas the Freundlich model (Eq. 5) accounts for heterogeneous, multilayer adsorption on surfaces with varied binding energies [68,69].

(4) $$1/q_{e}=1/Q_{0}+1/Q_{0}K_{L}C_{e}$$

(5) $$\text{ln}q_{e}=\text{ln}\;K_{F}+1/n\;\text{ln}\;C_{e}$$

Where *q_e_* ​represents the equilibrium adsorption capacity (mg/g), *C_e_* ​is the equilibrium concentration of the adsorbate (mg/L), *Q_0_* ​is the maximum adsorption capacity (mg/g), and *K_L_*​ is the Langmuir constant related to the affinity of binding sites (L/mg). In the Freundlich equation, *q_e_* ​is the equilibrium adsorption capacity (mg/g), *C_e_* ​is the equilibrium concentration of the adsorbate (mg/L), *K_F_* is the Freundlich constant indicating adsorption capacity, and 1/*n* is the heterogeneity factor reflecting the adsorption intensityIn contrast, Alg/HA/Chi was better described by the Freundlich model, indicating heterogeneous adsorption behavior and multilayer interactions arising from coexistence of carboxyl, amino, hydroxyl, and mineral-associated binding sites. This transition from predominantly Langmuir-type adsorption in simpler systems to Freundlich-type behavior in Alg/HA/Chi highlights how increasing structural complexity creates a wider spectrum of binding environments.

The isotherm parameters support these interpretations. The highest Q₀ for Alg (2521.15 mg/g) reflects its swollen porous network and abundant carboxylate groups. The lowest Q₀ for HA (263.12 mg/g) reflects rigid crystalline structure and limited accessibility. The intermediate values for Alg/HA (833.33 mg/g) and Alg/HA/Chi (1428.75 mg/g) demonstrate that adsorption is controlled by both site availability and accessibility. The increase in Q₀ following Chi incorporation indicates that introducing additional functional groups and improving network homogeneity enhances bioactive compound loading. The Kₗ values (affinity) follow a similar trend: Alg (0.32) > Alg/HA/Chi (0.29) > HA (0.02). The n values exceeding 1 in all cases confirm favorable, reversible adsorption. From a drug-delivery perspective, the enhanced loading capacity of Alg/HA/Chi suggests greater potential for sustained retention and controlled release of bioactive molecules—advantageous for wound-healing applications requiring prolonged therapeutic activity.

### 4.6 Kinetics of TE release

The distinct carrier-dependent release profiles demonstrate how matrix composition controls active compound liberation. Pure Alg exhibited slow, uniform release (∼30% after 30 days), reflecting the highly hydrated yet interconnected polymeric network where extract molecules are retained through physical entrapment and interactions with carboxylate groups. Diffusion pathways within the matrix remain restricted, making alginate an effective sustained-release carrier. Pure HA displayed burst release (∼59% within 7 days, ∼73% by day 30) due to weak surface adsorption and absence of a continuous polymeric network. The relatively homogeneous but limited adsorption sites result in weaker retention. Upon contact with release medium, surface-bound molecules are rapidly desorbed. The Alg/HA composite showed intermediate release (∼57% after 30 days), demonstrating how combining organic and inorganic phases fine-tunes release kinetics. The alginate matrix acts as a diffusion barrier, while HA increases structural heterogeneity and creates additional pathways for water penetration. The balance between diffusion control and matrix permeability produces a moderate release profile.

The Alg/HA/Chi system displayed the most complex, biphasic profile (∼68% in first phase, ∼86% total). This behavior is consistent with Freundlich isotherm adsorption, indicating heterogeneous distribution of adsorption energies and multilayer adsorption. The structurally diverse network contains binding sites with different affinities: weakly bound molecules release first, while strongly bound or deeply embedded molecules release more slowly. Such sequential release is advantageous for wound healing, providing both an initial therapeutic dose and prolonged delivery. The Korsmeyer–Peppas model [70] provided the best fit with highest R² values for all formulations, suggesting that extract release is governed by a combination of diffusion, matrix swelling, and structural relaxation of the polymer network. This is consistent with swelling results showing substantial fluid uptake by polymer-containing systems. The release behavior is directly linked to internal architecture, where porosity, polymer–polymer interactions, and polymer–extract interactions collectively regulate mass transport.

### 4.7 Biodegradation assessment

The two-stage degradation profiles reflect the complex interplay between water uptake, polymer relaxation, and microbial activity. The initial sharp increase (first 10 days) is due to rapid water uptake and swelling, facilitating polymer-chain relaxation and increasing accessibility of hydrolytic and microbial agents [71]. The temporary stabilization (days 10–15) may reflect the presence of HA or Chi, which reduce chain mobility and restrict diffusion pathways, acting as barriers against rapid degradation. The renewed increase after day 15 likely results from breakdown of key bonds due to accumulated enzymatic and microbial activity in soil. Newly formed pores and defects increase accessibility of internal regions to microorganisms and degradative agents. The slower degradation after day 25 as the system reaches a relatively stable state marks the transition to biological decomposition by soil microorganisms and partial dissolution of less stable or soluble phases.

Pure Alg exhibited the highest degradation (∼38% after 30 days) due to its hydrophilic nature, loosely organized network, and absence of reinforcing phases. The highly swollen structure facilitates rapid polymer disruption. Alg/HA showed the lowest degradation (∼24%) due to the crystalline, stable HA phase that restricts moisture and enzyme penetration. HA particles act as physical barriers, decreasing swelling-induced expansion and slowing transport of degradative agents. Alg/HA/Chi displayed intermediate degradation (∼27%), reflecting the formation of electrostatic interactions between chitosan amino groups and alginate carboxylate groups. Although chitosan itself is biodegradable, its incorporation strengthens the matrix and delays structural disintegration. This controlled degradation profile is advantageous for wound dressings requiring structural integrity during the healing period. The correlation between swelling and degradation is clear: materials with higher swelling capacity absorb more water, facilitating polymer-chain relaxation and increasing diffusion of microorganisms and degradative agents. Thus, the highly swollen Alg hydrogel exhibited the fastest degradation, while incorporation of HA and Chi slowed degradation by increasing structural cohesion and reducing accessibility

### 4.8 Hydrogel stability analysis

The stability test results demonstrate how composition affects structural integrity in aqueous environments. Pure Alg's rapid weight loss during the first 24 hours reflects the loose network structure where water molecules readily penetrate, hydrate polymer chains, and promote chain disentanglement [71]. Without reinforcing phases, soluble polymer fractions are released into the medium. The subsequent stabilization at ∼3.5% weight loss indicates a relatively stable condition after initial dissolution. The much lower and more stable weight loss profiles of Alg/HA and Alg/HA/Chi reflect enhanced structural stability. HA as a mineral phase strengthens the composite framework, reduces polymer chain mobility, and creates a more compact structure by filling pores. The particularly interesting behavior of Alg/HA/Chi, which exhibits minimal weight loss despite high swelling, demonstrates that chitosan strengthens the matrix through electrostatic interactions while maintaining porosity and hydrophilicity. Water penetration primarily results in hydrogel expansion rather than polymer loss. The combination of HA reinforcement and alginate–chitosan intermolecular interactions produces a structure that balances high water absorption with excellent dimensional and physicochemical stability. These stability characteristics are critical for wound dressings, where materials must maintain structural integrity during prolonged exposure to wound exudates while absorbing fluids and delivering therapeutic agents.

### 4.9 Antibacterial and antifungal evaluation

#### 4.9.1 Antibacterial activity

The antibacterial results demonstrate progressive enhancement through component incorporation, culminating in near-complete inhibition for the TE-loaded formulation. Pure Alg's modest antibacterial activity (45.44% for *B. cereus*, 25.98% for *A. tumefaciens*) is attributed primarily to physical characteristics rather than strong intrinsic bactericidal effects. The hydrated gel network creates an unfavorable environment for bacterial growth by limiting nutrient diffusion and bacterial attachment [72]. The negatively charged alginate surface may interact with bacterial membranes, leading to osmotic stress or membrane disruption—effects more pronounced in Gram-positive bacteria with thicker peptidoglycan walls. Residual metal cations or phenolic impurities from naturally extracted alginate may also contribute [71,73]. The marked enhancement upon HA incorporation (68.22% and 70.32%) reflects multiple mechanisms. HA introduces dispersed inorganic domains throughout the polymer network, increasing interfacial area available for microorganism–material interactions [74]. Ca²⁺-containing surfaces influence membrane integrity and cellular homeostasis [75]. The nanoscale topography of HA particles promotes direct contact-mediated damage. HA can promote ROS formation, damaging bacterial DNA, proteins, and membrane lipids [76]. Local pH alterations may further inhibit bacterial growth, and HA can act as an effective carrier for antimicrobial agents, allowing controlled release [77]. Alg/HA/Chi exhibited comparable antibacterial efficiency, confirming chitosan's synergistic role. The polycationic nature of chitosan—positively charged amino groups electrostatically interacting with negatively charged bacterial membranes—causes cell wall disruption, enhanced permeability, and leakage of cytoplasmic contents [78]. Chitosan can also penetrate bacterial cells, binding to DNA and vital enzymes, disrupting replication and metabolic processes. This polymer is particularly effective against Gram-negative bacteria due to their outer membrane sensitivity to cationic interactions [79]. The film-forming ability of chitosan prevents bacterial adhesion and biofilm formation [9].

Alg/HA/Chi/Ext demonstrated the highest antibacterial activity (98.99% against *B. cereus*, >74% against *A. tumefaciens*). FESEM and swelling studies indicate TE incorporation did not disrupt the hydrogel network but generated a porous, interconnected structure capable of accommodating and gradually releasing bioactive phytochemicals. The antimicrobial performance is associated with phenolic compounds (thymol, carvacrol) and the hydrogel architecture's ability to retain and deliver these compounds. These phytochemicals disrupt cell membranes, alter permeability, interfere with enzymatic activity, inhibit metabolic processes, and suppress protein and DNA synthesis [80]. Natural antioxidants in TE help stabilize bioactive compounds and maintain antimicrobial efficacy by reducing oxidative stress [81]. The higher activity against *B. cereus* compared to *A. tumefaciens* reflects structural differences. As Gram-positive, *B. cereus* lacks the outer lipopolysaccharide membrane characteristic of Gram-negative species, allowing easier penetration of hydrophobic phytochemicals. The outer membrane of *A. tumefaciens* acts as an additional permeability barrier, limiting diffusion and intracellular accumulation. Gram-negative bacteria generally exhibit greater intrinsic resistance to plant-derived phenolic agents. The MIC results corroborate the inhibition data. For *B. cereus*, MIC decreased from 62.5 µg/mL (Alg) to 3.9 µg/mL (Alg/HA/Chi/Ext). For *A. tumefaciens*, MIC decreased from 250 µg/mL to 3.66 µg/mL. The exceptionally low MIC values indicate the composite network serves as an efficient reservoir and delivery system for bioactive compounds.

#### 4.9.2 Antifungal activity

The antifungal results show a similar trend, with Alg/HA/Chi/Ext demonstrating the strongest response (99.12% against *A. flavus*, 56.34% against *F. avenaceum*, 91.22% against *F. solani*). MIC values below 5 µg/mL for all tested fungi, and remarkably low for *A. flavus* (0.59 µg/mL), outperforming amphotericin B. The superior antifungal efficacy is attributed to synergistic interaction between the hydrogel network and encapsulated TE phytochemicals. The porous structure facilitates water uptake and controlled diffusion of active compounds, while the reinforced alginate–chitosan–HA framework maintains structural stability during release. The antimicrobial behavior is governed by both intrinsic activity of components and network architecture that controls their accessibility, retention, and release.

#### 4.9.3 Limitations and future directions

It should be noted that *Thymus vulgaris* extract is a complex mixture of bioactive phytochemicals whose composition varies depending on plant origin, harvesting conditions, extraction procedure, and storage conditions. Consequently, antimicrobial activity and MIC values may differ among studies. Furthermore, the retention and recovery of individual phytochemical constituents after scaffold fabrication were not quantified. This represents a significant limitation, as absence of detailed compositional data prevents meaningful comparison of MIC values and release kinetics with literature reports and fundamentally limits reproducibility. Without chromatographic techniques (GC-MS or HPLC) to identify and quantify major bioactive compounds (thymol, carvacrol, other phenolics), the observed antimicrobial activity and release profiles cannot be directly attributed to specific compounds. Future studies employing chromatographic techniques are needed to: (i) quantitatively determine thymol and carvacrol content in both original extract and final hydrogel after fabrication; (ii) evaluate recovery efficiency of individual bioactive compounds after crosslinking, washing, and freeze-drying; (iii) correlate specific compound concentrations with antimicrobial efficacy and release kinetics; and (iv) standardize extract composition to ensure batch-to-batch reproducibility. These analyses are essential for meaningful comparison with literature data and eventual clinical translation.

## 5 Conclusion

This study successfully developed a novel Alg-Chi-HA-Ext. composite hydrogel with demonstrated superior properties for wound dressing applications. The key quantitative findings reveal: 1) Enhanced mechanical performance with TS increasing from 25.6 ± 5.5 MPa (pure Alg) to 50 ± 2.3 MPa, and toughness dramatically improving from 291.5 kJ/m³ to 1087.5 kJ/m³. Optimal swelling capacity of 766 ± 54%, comparable to pure Alg but with significantly better structural stability; 3) Excellent biocompatibility with cell viability reaching 88% after 72 hours in the MTT assay; 4) Potent antimicrobial activity showing 98.99% inhibition against B. cereus and 99.12% against A. flavus, with remarkably low MIC values below 5 µg/mL; and 5) Controlled release profile following the Korsmeyer-Peppas model. The incorporation of HA nanoparticles and TE created synergistic effects, where HA enhanced mechanical strength while TE contributed both antimicrobial properties and unexpected mechanical reinforcement. These quantitative results demonstrate that our composite hydrogel effectively balances mechanical integrity, swelling capacity, BC, and antimicrobial functionality, making it a highly promising candidate for advanced wound care, particularly for infected and chronic wounds that require multifunctional dressing solutions.

Overall, the developed Alg/HA/Chi/TE hydrogel demonstrated favorable structural, mechanical, swelling, biodegradation, cytocompatibility, controlled-release, and antimicrobial properties under laboratory conditions. The results suggest that the synergistic combination of alginate, chitosan, hydroxyapatite, and thyme extract may provide a useful platform for the development of multifunctional bioactive wound-dressing materials. However, several critical limitations must be acknowledged. The present study is limited to in vitro and physicochemical evaluations, and no sterilization or in vivo wound-healing assessments were performed. Therefore, further investigations are required to evaluate the biological performance, safety, and therapeutic efficacy of the developed hydrogel under clinically relevant conditions. Future studies should focus on sterilization validation (e.g., gamma irradiation, ethylene oxide, or ethanol-based methods), long-term stability, in vivo wound-healing models, and the optimization of formulation parameters to facilitate potential biomedical applications.

## CRediT authorship contribution statement

**Kimiya Karimi:** Writing – original draft, Resources, Methodology, Investigation, Formal analysis. **Sahebali Manafi:** Writing – review & editing, Validation, Supervision, Project administration, Conceptualization. **Fatemeh Mirjalili:** Writing – review & editing, Supervision, Data curation, Conceptualization.

## Declaration of competing interest

The authors declare that they have no known competing financial interests or personal relationships that could have appeared to influence the work reported in this paper.

## Data Availability

Data will be made available on request.
