# Supplementary material for: Development and characterization of a multifunctional alginate-chitosan hydrogel incorporating nano-hydroxyapatite and Thymus vulgaris for wound dressing applications
Source: Biomater Biosyst. 2026 Sep 3;23:100145. doi: 10.1016/j.bbiosy.2026.100145 (PMC13583980; doi:10.1016/j.bbiosy.2026.100145)
Supplement: Supplementary file 3 [file mmc3.docx]

Figure S3. Comparative diagram of MTT assay. The reported values are the *mean value* of 3 measurements (n=3), and error bars show the *standard deviations* (SD) calculated using one-way ANOVA analysis.
