# Supplementary material for: Development and characterization of a multifunctional alginate-chitosan hydrogel incorporating nano-hydroxyapatite and Thymus vulgaris for wound dressing applications
Source: Biomater Biosyst. 2026 Sep 3;23:100145. doi: 10.1016/j.bbiosy.2026.100145 (PMC13583980; doi:10.1016/j.bbiosy.2026.100145)
Supplement: Supplementary file 4 [file mmc4.docx]

Figure S4. (a) Release percentages of TE from pure and organic–inorganic composite matrices. Kinetic models fitted to TE release from different systems: (b) zero-order, (c) first-order, (d) Higuchi, (e) Hixson–Crowell, and (f) Korsmeyer–Peppas.
