# Supplementary material for: Development and characterization of a multifunctional alginate-chitosan hydrogel incorporating nano-hydroxyapatite and Thymus vulgaris for wound dressing applications
Source: Biomater Biosyst. 2026 Sep 3;23:100145. doi: 10.1016/j.bbiosy.2026.100145 (PMC13583980; doi:10.1016/j.bbiosy.2026.100145)
Supplement: Supplementary file 5 [file mmc5.docx]

Biodegradation rate (%)

| Time (Days) | Alg | Alg/HA | Al/HA/Chi |
| --- | --- | --- | --- |
| 5 | 9 | 8 | 7 |
| 10 | 17 | 14 | 16 |
| 15 | 20 | 16 | 19 |
| 20 | 29 | 21 | 24 |
| 25 | 35 | 23 | 26 |
| 30 | 38 | 24 | 27 |

Figure S5. Biodegradation of hydrogel systems in soil as a function of time.
