# Supplementary material for: Development and characterization of a multifunctional alginate-chitosan hydrogel incorporating nano-hydroxyapatite and Thymus vulgaris for wound dressing applications
Source: Biomater Biosyst. 2026 Sep 3;23:100145. doi: 10.1016/j.bbiosy.2026.100145 (PMC13583980; doi:10.1016/j.bbiosy.2026.100145)
Supplement: Supplementary file 6 [file mmc6.docx]

Weight loss (%)

| Time (h) | Alg | Alg/HA | Al/HA/Chi |
| --- | --- | --- | --- |
| 0 | 0 | 0 | 0 |
| 6 | 1.12 | 0.45 | 0.46 |
| 12 | 2.38 | 0.66 | 0.76 |
| 24 | 3.55 | 0.78 | 0.87 |
| 48 | 3.6 | 0.79 | 0.89 |
| 72 | 3.71 | 0.8 | 0.92 |

Figure S6. Stability test results of the studied samples in distilled water.
