# Supplementary material for: Development and characterization of a multifunctional alginate-chitosan hydrogel incorporating nano-hydroxyapatite and Thymus vulgaris for wound dressing applications
Source: Biomater Biosyst. 2026 Sep 3;23:100145. doi: 10.1016/j.bbiosy.2026.100145 (PMC13583980; doi:10.1016/j.bbiosy.2026.100145)
Supplement: Supplementary file 7 [file mmc7.docx]

Figure S7. The antimicrobial activity of the synthesized HGs: (a) bacterial growth inhibition percentage and (b) MIC values of HGs against *B. cereus* and *A. tumefaciens*; (c) fungal growth inhibition percentage and (d) MIC values against *A. flavus*, *F. avenaceum*, and *F. solani*. Data are expressed as mean ± standard deviation (N = 3). *P < 0.001 vs. Alg; ###P < 0.001 vs. Alg/HA; and $P < 0.05 vs. Alg/HA/Chi.
