# Supplementary material for: Development and characterization of a multifunctional alginate-chitosan hydrogel incorporating nano-hydroxyapatite and Thymus vulgaris for wound dressing applications
Source: Biomater Biosyst. 2026 Sep 3;23:100145. doi: 10.1016/j.bbiosy.2026.100145 (PMC13583980; doi:10.1016/j.bbiosy.2026.100145)
Supplement: Supplementary file 8 [file mmc8.docx]

Table S1. Mechanical parameters derived from tensile testing of the investigated samples (The reported values are the mean ± standard deviation (SD) values of three distinct tests)

| Sample | UTS (MPa) | Young’s Modulus (MPa) | Maximum Strain (%) | Toughness (kJ/m³) |
| --- | --- | --- | --- | --- |
| Alg | 25.6±5.5 | 16.2±0.95 | 2.2±0.27 | 291.5 |
| Alg/HA | 11.88±1.45 | 11.7±0.8 | 1.27±0.17 | 75.43 |
| Alg/HA/Chi | 20.86±2.2 | 12.5±1 | 1.96±0.12 | 204 |
| Alg/HA/Chi/Ext | 50±2.3 | 16±1.5 | 4.35±0.55 | 1087.5 |
