# Supplementary material for: Development and characterization of a multifunctional alginate-chitosan hydrogel incorporating nano-hydroxyapatite and Thymus vulgaris for wound dressing applications
Source: Biomater Biosyst. 2026 Sep 3;23:100145. doi: 10.1016/j.bbiosy.2026.100145 (PMC13583980; doi:10.1016/j.bbiosy.2026.100145)
Supplement: Supplementary file 9 [file mmc9.docx]

Table S2. Thermodynamic parameters obtained from Langmuir and Freundlich adsorption isotherms for the investigated samples.

| K_F_ | n | Q_0_ (mg/g) | K_L_ | Sample |
| --- | --- | --- | --- | --- |
| 27.18 | 1.99 | 2521.15 | 0.32 | Alg |
| 2.71 | 1.15 | 263.12 | 0.02 | HA |
| 18.65 | 1.31 | 833.33 | 0.15 | Alg/HA |
| 21.33 | 1.27 | 1428.75 | 0.29 | Alg/HA/Chi |
